# Youth mental health in the time of COVID-19

**DOI:** 10.1017/ipm.2020.84

**Published:** 2020-07-02

**Authors:** E. Power, S. Hughes, D. Cotter, M. Cannon

**Affiliations:** 1 Department of Psychiatry, Royal College of Surgeons in Ireland, Smurfit Building, Beaumont Hospital, Beaumont Road, Dublin 9, Ireland; 2 Union of Students of Ireland, 14 Mount Street Upper, Dublin 2, Ireland; 3 Department of Psychiatry, Beaumont Hospital, Dublin 9, Ireland; 4 Trinity College Institute of Neuroscience, Lloyd Building, Trinity College Dublin, Dublin 2, Ireland

**Keywords:** COVID-19, digital, youth mental health

## Abstract

Youth mental health is a rapidly developing field with a focus on prevention, early identification, treatment innovation and service development. In this perspective piece, we discuss the effects of COVID-19 on young people’s mental health. The psychosocial effects of COVID-19 disproportionately affect young people. Both immediate and longer-term factors through which young people are affected include social isolation, changes to the delivery of therapeutic services and almost complete loss of all structured occupations (school, work and training) within this population group. Longer-term mechanisms include the effects of the predicted recession on young people’s mental health. Opportunities within this crisis exist for service providers to scale up telehealth and digital services that may benefit service provision for young people’s mental health in the future.

## Introduction

Mental disorders are the largest cause of years lived with disability worldwide (Whiteford *et al.* 2015). Up to 80% of mental disorders first occur before the age of 26 (Caspi *et al.* 2020; Kessler *et al.* 2005). Earlier age of onset of mental disorder is associated with increased risks of development of comorbidity and persistence of mental health disorder to midlife (Caspi *et al.* 2020). Young people who remain free of mental disorder have longitudinally better outcomes (Caspi *et al.* 2020). Youth mental health problems cast a long shadow over adult health and psychosocial functioning. The magnitude of the effects of mental health problems in youth over the life course far surpasses the effects of early physical health problems (Goodman *et al.* 2011). In this paper, we will outline how youth, whilst less susceptible to severe COVID-19 infection, is more at risk of the negative psychosocial effects of the pandemic that was officially declared on the 11th of March 2020 (Holmes *et al.* 2020).

## Disrupted transitions

Entering the labour force from education marks one of the most significant transitions that takes place during a young person’s life, and this transition has become more complex in recent decades (Arnett 2000). During recent periods of economic recession, young people have much higher rates of unemployment (Bell and Blanchflower, 2011). The effects of periods of unemployment in youth have disproportionate and long-lasting effects on income and health beyond the period of economic recession as well as risks of concurrent and future insecure employment (Kahn, 2010; Cockx, 2016). Currently, a majority of young people (51%) between the ages 15–24 within the labour force (i.e. those available for work and not in education) are unemployed in Ireland (CSO, 2020). This represents almost 2.5 times the unemployment rate in adults and almost 2.5 times the peak unemployment rate in the same age reference category during the most recent economic recession (CSO, 2020). Analysis from the Economic and Social Research Institute predicts Ireland to experience a severe economic recession in the coming year (McQuinn *et al.* 2020). In periods of recession, more highly educated youths have moderate long-lasting reductions in income for at least a period of 10 years, whilst losses are restored for lower educated youths more quickly (Cockx, 2016). Graduating from university during a recession has particular long-lasting and large impacts on earning potential (Kahn, 2010). During periods of recession, high educational level may not be a protective for young people’s mental health and may be a risk factor for poor mental health outcomes specifically when youth are engaged in insecure working arrangements or are unemployed. One recent study from Italy suggests that young highly educated women are at most increased risk of poorer mental health due to economic insecurity during periods of recession (Fiori *et al.* 2016). Economic inactivity (i.e. not being in employment education or training) increases risks of suicidal thoughts and behaviours in young people beyond the effects of prior mental health vulnerability (Power *et al.* 2015). The mental health effects of unemployment in youth persist to midlife with those exposed to unemployment in youth having increased rates of common mental health symptoms like anxiety and depression on long-term follow-up (Virtanen *et al.* 2016).

Youth is also a point of cognitive, social and emotional transitions. Young people, particularly adolescents, have different cognitive approaches to making social decisions in comparison to adults (Blakemore & Choudhury, 2006). Social connectedness and social identity have more prominence in youth and high rates of reported loneliness are reported in young people (Matthews *et al.* 2019). Loneliness in young adults is associated with a number of negative health behaviours and indicators of poor mental health independent of other risk factors (Matthews *et al.* 2019). In this context, young people may be more affected by the negative psychosocial consequences of ‘lockdown’ and social distancing than adults. Young people may also find it more difficult to cope with the current crisis as their coping skills are not equivalent to that of a fully-fledged adult as coping is a developmentally acquired skill (Fields & Prinz, 1997).

## An acute on chronic public health crisis

Investing in early intervention in mental health has potential to reduce population level chronic disease morbidity. Early intervention programmes in psychosis show reductions in mortality and improvements in significant and pragmatic indicators of psychosocial functioning (McGorry, 2015; Pollard *et al.* 2020). Despite these innovations, new service models for psychosis are only selectively available to a small proportion of the population depending on geographical area. Early intervention for other specific serious mental disorders is in earlier phases of development (Chanen *et al.* 2017; Vieta *et al.* 2018). Early intervention services for adolescents and young people at primary care level (such as Jigsaw in Ireland and Headspace in Australia) are also being rolled out in line with international best practice (Hetrick *et al.* 2017; McGorry *et al.* 2013). Despite new and evolving evidence in youth mental health, resource allocation for young people’s mental health remains insufficient. A recent survey of Child and Adolescent Mental Health Services consultants showed high levels of burnout (McNicholas *et al.* 2020). Known precipitants to this are insufficient staffing, incomplete coverage and long waiting lists (McNicholas, 2018). Incomplete coverage is a specific issue for primary care services such as Jigsaw. In this current pandemic crisis, educational, health and social care services have had to curtail the level of service offered to young people and their families. This COVID-19 pandemic presents an ‘acute on chronic crisis’ for services for young people where demand on services is likely to increase but supply of services is further constrained and inconsistent.

Ensuring the material needs and physical health of communities is the immediate priority in any public health emergency, conflict situation or natural disaster. The mental health needs of young people can be overlooked in a public health crisis (Danese *et al.* 2020). There are worries for a ‘final wave’ of the effects of the virus in terms of the negative mental health and social consequences borne by young people whom have little control over their environmental circumstances. There are many potential adverse consequences for young people who have lost access to structured school and college and work environments. There are broad physical and mental health implications for all young people. Negative physical health consequences such as poorer sleep, poorer diet, increased sedentary behaviour and loss of cardiometabolic fitness are more common and these are likely to relate to poorer mental health during COVID (Wang *et al.* 2020). However, the mental health consequences may be more significant and long lasting. Early survey reports from China highlight the negative mental health consequences of exposure to the pandemic in young people, reporting increased rates of anxiety, poorer sleep and irritability (Jiao *et al.* 2020).

The mental health impacts of any disaster are unevenly distributed. Those with lower social capital and those in vulnerable positions are most at risk. One example is young people in temporary accommodation or direct provision. These groups of individuals and their family members face an already higher burden of mental health risk as well as direct increased risks of COVID infection due to unsuitable accommodation (Rosenthal *et al.* 2020). Reports of increased rates of exposure to domestic violence are concern for vulnerable young people also (Gunnell *et al.* 2020; Chandan *et al.* 2020). Public institutions buffer the effect, length and severity of childhood adversity and trauma. This is through a multitude of mechanisms such as providing free school meals, providing a physically safe environment for part of the day through school participation, support through voluntary services and mandated child welfare/protection reporting. Access to a supportive adult is a protective factor for a young person’s mental health and some will have lost this protective factor during this crisis through loss of supports outside the family home (Dooley *et al.* 2015). In this context, prioritizing equity in reopening services is important. Services, such as school placements, should be provided for the most vulnerable young people first.

Early research efforts from the YoungMinds organisation in the UK highlight the predominance of concerns around the psychological and social consequences of the pandemic response, particularly on young people (YoungMinds, 2020; Holmes *et al.* 2020). In this recent UK survey, 83% of young people with mental health needs believed that COVID-19 had an adverse impact on their mental health, with specific concerns around loss of social contact and structured activities. In this survey, young people with varying types of mental health conditions, such as obsessive compulsive disorder, anxiety disorders and anorexia nervosa specifically noted that the crisis had worsened their pre-existing conditions. One in four young people whom had been accessing mental health supports prior to the pandemic reported that they no longer had access because of the crisis (YoungMinds, 2020).

## Research priorities

The effects of mandated self-isolation in terms of morbidity and mortality of young people most at risk of negative health consequences of the pandemic should be a primary focus of research. Existing cohort studies should be leveraged to investigate the changes in health status in youth during the time of COVID (Holmes *et al.* 2020). The World Health Organisation recommends that the mental health needs of young people should be included within coordinated statutory disaster response mechanisms through at minimum the dissemination of psychoeducational and self-help resources for young people to promote universal advice on maintaining positive health behaviours (World Health Organisation, 2020). Simple guidance on addressing young people’s concerns with age appropriate emotion-focused language is highly likely to be significantly effective (Dalton *et al.* 2020). Guidance for caregivers on the positive impact of maintaining their own well-being is also important. Young people’s mental health is strongly influenced by the well-being of their caregivers. Evidence-based digital platforms, such as interventions targeting disruptive behaviour in children, may benefit in being rolled out at this time. They are effective in reducing target symptoms and secondary care utilisation after 2-year follow-up (McGrath *et al.* 2013; Sourander *et al.* 2018).

## Opportunities for change

Opportunities for positive change exist at many levels, for some individual young people, for families, for health and social care professionals and researchers, for communities and for specific sectors. Many young people will have unique opportunities to spend more time with their families and a small minority (8%) in the YoungMinds survey reported this (YoungMinds, 2020). For caregivers working from home or temporarily furloughed, current remote working arrangements could offer opportunities for longer-term flexible working conditions that are common in many Nordic countries and appear to have a well-being dividend for young people and their families (Caan & Jenkins 2008). The current crisis may also be an opportunity to investigate the effects of community cohesion on prosocial behaviours, psychopathology and suicidality in young people (Oosterhoff *et al.* 2020).

Opportunities exist for health care professionals to change work practices to rapidly scale up effective digital and digital-hybrid interventions. Whilst digital interfaces in mental health can be effective, clinician resistance is cited as hindering widespread uptake (Wind *et al.* 2020). Telepsychiatry is broadly acceptable to a large majority of young people with severe mental disorders such as psychosis, with some caveats around potentially increased dropout from treatment (Lal *et al.* 2020). Digital platform development is specifically important for youth as young people are digital natives who look online first for information related to their mental health. Emerging platforms offer partially guided adaptations of standard therapies (such as cognitive behavioural therapy) through video games (Chapman *et al.* 2016). These are scalable, effective and youth-friendly alternatives to traditional therapies. Emerging digital services offer a democratisation of access to emerging and specialist therapies, for example, in depression and psychosis in young people (Rice *et al.* 2018; McEnery *et al.* 2019). Digital services have many practical advantages, as access to services is contingent on geography, particularly in Ireland. One positive outcome of the pandemic is that clinicians and patients have had the opportunity to use digital or tele-platforms where previously this option would not have been available to them. Digital platforms have potential to improve quality of care particularly in non-urban areas where there may be no or insufficient access to services locally. Digital services may also reduce costs associated with obtaining mental health care for young people and their caregivers.

In conclusion, we have discussed why the COVID-19 pandemic will disproportionately affect young people both in the short- and long-term, and why the harms of the pandemic at a population level are inequitably distributed. The COVID-19 pandemic will however be a catalyst to rethink the delivery of services and to provide more accessible, equitable and efficient services in the future.
